# Comparison of Conventional and Molecular Diagnostic Techniques for Detection of *Blastocystis* sp. in Pig Faeces

**Published:** 2018

**Authors:** Tamás SÜLI, Gordana KOZODEROVIĆ, Aleksandar POTKONJAK, Stanislav SIMIN, Verica SIMIN, Vesna LALOŠEVIĆ

**Affiliations:** 1. Dept. of Veterinary Medicine, Faculty of Agriculture, University of Novi Sad, Novi Sad, Serbia; 2. Prophyl Animal Health Ltd, Mohács, Hungary; 3. Faculty of Education, University of Novi Sad, Novi Sad, Serbia; 4. Pasteur Institute, Novi Sad, Serbia

**Keywords:** *Blastocystis*, Pig, PCR, In vitro culture, Sensitivity

## Abstract

**Background:**

*Blastocystis* is a common protist colonizing the gastrointestinal tract of humans and various animals. Pigs have been suggested to be a reservoir for human *Blastocystis* infections because of high prevalence of the parasite in these animals and the presence of zoonotic subtypes. Nevertheless, epidemiological data is often misinterpreted due to the lack of standard diagnostic procedures. This study aimed to compare the sensitivity of different diagnostic techniques in detection of *Blastocystis* sp. in pigs.

**Methods:**

Overall, 48 individual faecal samples were collected from pigs reared in an intensive farming system (Autonomous Province of Vojvodina, Serbia) and were tested by microscopic examination of direct wet mount, in vitro cultivation in modified Jones’ medium and conventional PCR for rRNA gene.

**Results:**

Xenic in vitro cultivation in Jones’ medium showed higher sensitivity than direct wet mount when we compared it with PCR. Namely, the estimated sensitivity of direct wet mount was 46.15%, while the sensitivity of in vitro cultivation was 84.62%.

**Conclusion:**

Low sensitivity of conventional parasitological compared to molecular methods is proven. Thus, reports on prevalence that rely solely on microscopy of faecal samples (unprocessed or concentrated) are probably underestimating the true prevalence of the parasite.

## Introduction

*Blastocystis* is a unicellular microorganism found in the gastrointestinal tract of humans and various animals (1, 2). *Blastocystis* has a worldwide distribution and presents a frequent finding in different parasitological surveys. *Blastocystis* is beside humans, isolated from amphibians, reptiles, insects, many species of birds and mammals, zoo animals, especially primates. In addition to wild-life and zoo animals, *Blastocystis* is found in domestic animals like pigs, poultry, ruminants and horses (3).

Although not yet fully clarified, the close contact of humans and pigs, whether we speak about intensive farming or small rural households, represents a substantial risk of zoonotic transmission of *Blastocystis*. The nature of the parasite and the lack of standard diagnostic procedures led to a number of perplexities and sometimes to completely wrong interpretation of the obtained data. The traditional diagnostic method and a primary choice in the diagnosis of *Blastocystis* in many laboratories is the microscopy of direct faecal smear. However, this method has significant limitations. Microscopy of fresh faecal material in native form can be challenging, even for the most experienced laboratory personnel. Xenic in vitro cultivation (XIVC) is a simple and low-cost method for detection of *Blastocystis*.

Nevertheless, for understanding the epidemiology of the parasite and the role of animals in human blastocystosis, the use of molecular diagnostic methods is inevitable. A number of studies have compared performances of different diagnostic approaches (4–8). Authors generally report low sensitivity of traditional techniques like direct faecal smear and formol ethyl acetate concentration technique (FECT) when compared to molecular detection (PCR) and XIVC. The diagnostic procedures were mainly tested on human faecal specimens and to our knowledge, there is limited data about different diagnostic techniques when animals are the subject of the investigation.

The aim of our study was to compare the sensitivity of different diagnostic techniques in detection of *Blastocystis* sp. in pig faeces.

## Materials and Methods

### Direct wet mount and in vitro cultivation

Overall, 48 individual faecal samples were collected from pigs reared in an intensive farming system. Faecal samples were collected in the period August–November 2016, from pig farms that are in the ownership of the most relevant swine producing companies in the Autonomous Province of Vojvodina (Serbia). All companies voluntarily consented to collaborate in this study. All samples were delivered to the laboratory within three hours after sampling and were processed immediately. From each faecal specimen one aliquot of approximately 300 mg was frozen on −20 °C in microcentrifuge tubes until the DNA extraction. In the process of xenic (non-sterile) in vitro cultivation, approximately 50–100 mg of each sample was inoculated into modified Jones’ medium supplemented with 10% horse serum (9). The cultures in centrifuge tubes with screw cap were incubated at 37 °C for 48–72 h. The sediment was examined under both the low (× 10) and high power (× 40) objectives. Iodine stain was used for the more distinctive visualization of cellular structures. The remaining faecal material was thoroughly homogenized with a wooden applicator stick. Approximately 2 mg of faeces was emulsified on a glass slide in one drop of physiologic saline and covered with a coverslip. As in the case of in vitro cultivation, direct wet mount preparations were examined under both low (× 10) and high power (× 40) objectives.

### DNA extraction

Aliquots of 180–220 mg of frozen faecal material were weighed into sterile microcentrifuge tubes placed on ice. Genomic DNA extraction from *Blastocystis* cells in faecal samples was performed using QIAamp DNA Stool Mini Kit (QIAGEN) following the manufacturer’s instructions.

### PCR amplification

A PCR protocol (10) was employed to amplify the 600 bp region of the small subunit ribosomal RNA gene of *Blastocystis* using the genomic DNA extracted from pigs’ faecal samples.

Primers BhRDr (*5*′–GAGCTTTTTAACTGCAACAACG–*3*′) and RD5 (*5*′–ATCTGGTTGATCCTGCCAGT–*3*′) were used. The composition of the PCR reaction was: 10 μl of HotStar-Taq *Plus* Master Mix (Qiagen), 0.25 μl of each 100μM primer, 2 μl of Coral Load Concentrate and 2 μl of genomic DNA in a final volume of 20 μl. PCR amplification was performed in a thermocycler (Techne TC-412) under the following conditions: initial denaturation at 95 °C for 5 min, 30 amplification cycles of 1 min each was carried out at 94, 59 and 72 °C, with an additional 2 min final extension. PCR products were kept at 4 °C until used. PCR products were electrophoresed in 2% agarose gels with Tris-Borate-EDTA (Gibco^®^ UltraPure^TM^ TBE Buffer) electrophoresis buffer. Molecular weight marker (ThermoScientific GeneRuler 100bp Plus DNA Ladder) was included in each run. Bands were analyzed using a UV gel documentation system (Serva BlueCube 300) after ethidium bromide staining.

### Statistical analysis

The sensitivity of direct wet mount and *in vitro* cultivation was calculated using Epitools epidemiological calculators (AusVet Animal Health Services and Australian Biosecurity Cooperative Research Centre for Emerging Infectious Disease, http://epitools.ausvet.com.au). The desired level of confidence was 0.95 and the results are presented as estimates of sensitivity with specified Clopper-Pearson (exact) confidence limits.

### Ethical considerations

All procedures were carried out in accordance with the requirements of the national law on the animal welfare (Official Herald of Republic of Serbia No.41/2009). The law is in compliance with the corresponding directives of the European Parliament and the Council of the European Union.

## Results

**Fig. 1:**
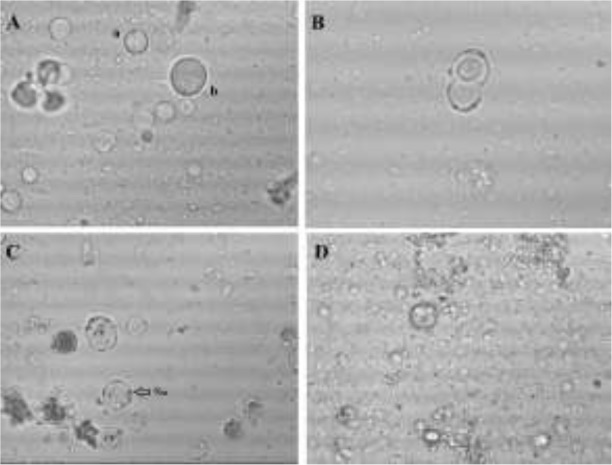
*Blastocystis* in culture and fresh faecal material. A: vacuolar (a) and granular forms (b) of *Blastocystis* sp. in culture. B: cells undergoing binary fission (in culture) C: cells in culture with clearly visible nuclei (Nu) and central vacuole (CV) after iodine staining. D: *Blastocystis* in fresh faecal material

**Fig. 2:**
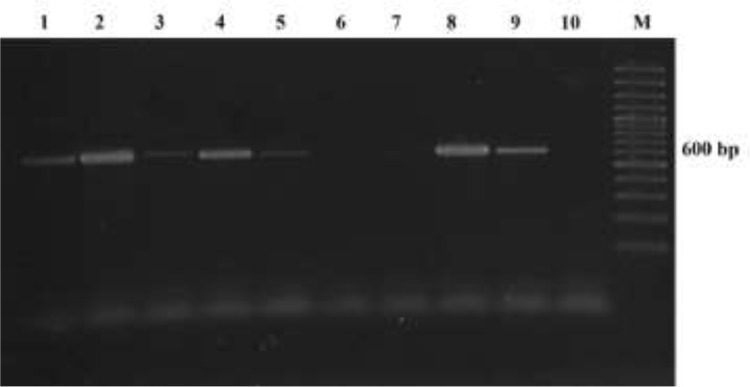
Ethidium bromide stained 2% agarose gel of PCR products of *Blastocystis* sp. from pigs. M, molecular marker (100 bp); lanes 1, 2, 3, 4, 5 and 8, positive samples; lanes 6 and 7, negative samples; lane 9, positive control; lane 10, negative control

Out of 48 samples, 21 (43.75%) samples showed positive by direct faecal smear examination. The most common findings were granular and cyst forms of *Blastocystis*, while the vacuolar forms were present in a smaller proportion. No amoeboid forms were visible in direct wet mounts. Besides *Blastocystis* sp., the direct faecal smear examination showed the presence of *Giardia* sp., *Balantidium coli,* and *Eimeria* sp. in a small number of samples. Among 48 samples, 38 (79.17%) were positive by xenic in vitro cultivation in Jones’ medium. The most common forms of *Blastocystis* in Jones’ medium were vacuolar and granular forms. After iodine staining, the cellular structures as the central vacuole, band of cytoplasm, nuclei and surface coat were clearly distinguishable (Fig. 1). PCR reactions were considered positive if a band of 600 bp was visible in the gel (Fig. 2). Using PCR detection from faeces, we found 39 (81.25%) samples positive for *Blastocystis* sp. The results of different diagnostic methods are summarized in Table 1. Three diagnostic methods were in agreement in 22 cases, while in all other cases some disagreements were found. There were disagreements in 24 cases when we compared direct wet mount and PCR, with negative optical microscopy samples that were positive to PCR and vice. Moreover, disagreements were found in 11 cases when we compared in vitro cultivation in Jones’ medium with PCR (Table 2).

**Table 1:** Results of different diagnostic methods in detecting *Blastocystis* sp.

***Method***	***Number of positive samples***	***Number of negative samples***
Direct wet mount	21	27
Xenic in vitro cultivation	38	10
PCR	39	9

**Table 2:** Positive and negative samples to *Blastocystis* sp. using different diagnostic methods

		***PCR***	
		***Positive***	***Negative***
Direct wet mount	Positive	18	3
	Negative	21	6
In vitro cultivation	Positive	33	5
	Negative	6	4

Compared to PCR, the estimated sensitivity of direct wet mount was 46.15% (lower 95% ±: 0.3009 and upper 95% ±: 0.6282), while the sensitivity of in vitro cultivation was 84.62% (lower 95% ±: 0.6947 and upper 95% ±: 0.9414) when we compared it with PCR.

## Discussion

Pig production is an important part of the economy of many countries, and one of the major livestock branches of the global economy. Among the top five pig producing countries, there are three developing countries: Vietnam, Brazil, and China. Roughly 50% of the world’s pig population is in China. Domestic pigs and wild boars are susceptible to a large number of infectious and parasitic diseases. Some of these diseases are limited to pigs, while other diseases are easily shared among pigs and various wild and domestic animals. As the number and geographic distribution of domestic pigs and wild boars are constantly growing, it will increase the number of contacts among pigs and other domestic animals, and consequently will increase the probability of direct or indirect exposure of people to various parasites of pigs. *Blastocystis* is one of the parasites of pigs, which has the potential to infect humans, therefore, it is reasonable to include it in the risk analysis of zoonotic transmission. *Blastocystis* infects significantly more people in developing countries than developed countries (1). If we take the statistical data into account according to which more than 70% of the world population of pigs is located in the developing part of the world, and different reports of high rate of infection with *Blastocystis* in pigs, it is reasonable to focus research on the role of pigs in the epidemiology of human blastocystosis.

Numerous studies are published on the prevalence of *Blastocystis* in humans worldwide. The reported prevalence ranges between 0.5% and 62%. The prevalence may be low in countries such as Japan (0.5%–1%) (11, 12) and Singapore (3.3%) (13) and high in countries such as Argentina (27.2%) (14), Brazil (40.9%) (15), Cuba (38.5%) (16), up to 100% in the children of Senegal River Basin (17). *Blastocystis* is one of the most common eukaryote reported to colonize humans in Iran (18, 19). There are several reports on *Blastocystis* infection and prevalence in pigs. The first description of infection of pigs with *Blastocystis* sp. was given by in 1976 (20). The presence of *Blastocystis* was determined in pigs using in vitro cultivation in a medium with horse serum, ranging from three-day-old piglets to adult pigs and reported a prevalence of 68–93% (21). Faecal samples from pigs from 17 farms were analyzed in Aragon (Spain) (22). Faecal specimens were concentrated by a modified formalin-ethyl acetate sedimentation method and examined by light microscopy using wet and iodine-stained preparations. In this study *Blastocystis* sp. was identified in the faeces of 27 pigs, representing a prevalence of 7.5%.

Overall, 395 pig faecal samples were collected from 11 intensive growing systems from the Valencian Community (Spain) (23). The authors investigated faecal samples from farmed pigs by optical microscopy and PCR. A global prevalence of 46.8% was observed (23). Faecal samples were analyzed using PCR from pigs and in-contact humans from commercial intensive piggeries in Southeast Queensland (Australia) and a village in rural Cambodia (24). The prevalence of *Blastocystis* in Australian and Cambodian pigs was 76.7% and 45.2%, respectively. The reported prevalence data greatly varies and must be interpreted with caution.

The laboratory diagnosis of *Blastocystis* can be quite challenging and the prevalence data can be easily influenced, in a positive or negative manner, depending on the method of choice for the diagnosis of the parasite. As with most parasites, for the detection of *Blastocystis,* both direct and indirect diagnostic methods are developed. Direct methods are based on the morphology (microscopy) and the detection of DNA (PCR) or antigen (immunofluorescence, ELISA), while the indirect methods are generally based on detection of the antibodies (25). Despite being the first choice in the diagnosis of *Blastocystis* worldwide, the usefulness of the direct microscopic examination is limited in clinical microbiology laboratories and epidemiological studies. The polymorphic nature of the organism in wet mounts can result in confusion with other organisms or inanimate formations (26). There are no exact determinants to identify vacuolar, granular, avacuolar, multi-vacuolar, amoeboid and cyst forms, and there is a possibility that some of these forms are artifacts resulting from various factors, e.g. the presence of oxygen (27). Our study demonstrated a low sensitivity of direct wet mount, which was 46.15% when we compared it with PCR. Trichrome staining is a routine technique used in many microbiological laboratories. Studies have shown that trichrome staining is more sensitive for detection of intestinal protozoa, including *Blastocystis* than direct and iodine-stained wet mounts (28, 4). Another commonly used technique in laboratory diagnosis of *Blastocystis* is formol ethyl acetate concentration technique (FECT). FECT results in very poor sensitivity and should be discouraged in laboratory diagnosis of *Blastocystis* (29, 6, 7). Xenic in vitro culture (XIVC) is a method of cultivation in the presence of an unidentified bacterial flora. *Blastocystis* can grow and reproduce in different xenic cultures (30, 31). Due to its simplicity and low cost, Jones’ medium is popular in the detection and maintenance of *Blastocystis* culture. When the subject of investigation is human faecal samples, cultivation in Jones’ medium has a sensitivity between 52% and 79% compared to real-time PCR protocols (32, 33). When we compared in vitro cultivation and conventional PCR which amplified DNA extracted directly from pig faeces, the sensitivity of XIVC was 84.62%.

All three diagnostic techniques were in agreement in 22 cases. In all other samples, one or more disagreements were found. The most frequent disagreement (15 samples) was a negative direct wet mount and positive results of in vitro cultivation and PCR. Microscopy of direct wet mounts requires certain skills and experience. *Blastocystis* cells in native preparations can be so rare and inconspicuous that they can be simply overlooked even by an experienced laboratory technician. Moreover, morphological forms of *Blastocystis* identified unambiguously, like the vacuolar form, may not predominate in fresh faecal specimens. Other factors such as irregular shedding of the parasite from the host or time elapsed from sampling to examination, can also contribute to the low sensitivity of direct wet mount. Six samples were negative by direct wet mount and in vitro cultivation but positive by PCR. Possibly, the faecal samples contained a low number of parasites missed during microscopy of direct wet mounts and did not contain viable cells that could by amplified with in vitro cultivation. Three samples were positive by wet mount and XIVC, but negative by PCR. A possible explanation for this result is an inefficient DNA extraction from the samples. Two samples were positive only by in vitro cultivation, while wet mount and PCR showed negative results. A low number of cells and insufficient extracted DNA could be possible explanation for this result. Besides efficient DNA extraction, choosing adequate primer sets and PCR protocol are crucial for efficient molecular diagnosis. A number of tools have been developed for molecular analysis of *Blastocystis*. Two approaches for typing are widely used, one of them uses sequence-tagged-site (STS) primers, while the other one involves analysis of SSU rDNA variation. The barcode region of SSU rDNA gene (10) has been validated as a marker of overall genetic diversity of *Blastocystis* (34).

In this study, we used primers BhRDr and RD5 for the detection of *Blastocystis* in pigs’ faeces. The PCR protocol showed high sensitivity when compared with traditional parasitological methods, and should be encouraged in diagnosis of *Blastocystis*. The use of BhRDr and RD5 primers enables typing of *Blastocystis* and further exploration of molecular epidemiology of this parasite.

## Conclusion

Data on prevalence of *Blastocystis* in pigs, or any other species, should be interpreted with caution, particularly if just one diagnostic approach was employed. Reports on prevalence of *Blastocysis* in pigs that rely solely on microscopy of faecal samples (unprocessed or concentrated) are probably underestimating the true prevalence of the parasite. In this study, xenic in vitro cultivation in Jones’ medium showed relatively high sensitivity. Short term (48–72 h) XIVC is cost-effective and easy to use and should be encouraged in routine diagnostic and screening. Modern molecular approaches should be used to clarify the role of pigs in the zoonotic transmission of *Blastocystis*.
